# A Comparison of Non-Typhoidal *Salmonella* from Humans and Food Animals Using Pulsed-Field Gel Electrophoresis and Antimicrobial Susceptibility Patterns

**DOI:** 10.1371/journal.pone.0077836

**Published:** 2013-10-30

**Authors:** Carol H. Sandt, Paula J. Fedorka-Cray, Deepanker Tewari, Stephen Ostroff, Kevin Joyce, Nkuchia M. M’ikanatha

**Affiliations:** 1 Bureau of Laboratories, Pennsylvania Department of Health, Exton, Pennsylvania, United States of America; 2 United States Department of Agriculture, Agricultural Research Service, Athens, Georgia, United States of America; 3 Division of Infectious Disease Epidemiology, Pennsylvania Department of Health, Harrisburg, Pennsylvania, United States of America; 4 Pennsylvania Veterinary Laboratory, Pennsylvania Department of Agriculture, Harrisburg, Pennsylvania, United States of America; 5 National Antimicrobial Resistance Monitoring System, Centers for Disease Control and Prevention, Atlanta, Georgia, United States of America; The University of Hong Kong, Hong Kong

## Abstract

Salmonellosis is one of the most important foodborne diseases affecting humans. To characterize the relationship between *Salmonella* causing human infections and their food animal reservoirs, we compared pulsed-field gel electrophoresis (PFGE) and antimicrobial susceptibility patterns of non-typhoidal *Salmonella* isolated from ill humans in Pennsylvania and from food animals before retail. Human clinical isolates were received from 2005 through 2011 during routine public health operations in Pennsylvania. Isolates from cattle, chickens, swine and turkeys were recovered during the same period from federally inspected slaughter and processing facilities in the northeastern United States. We found that subtyping *Salmonella* isolates by PFGE revealed differences in antimicrobial susceptibility patterns and, for human *Salmonella*, differences in sources and invasiveness that were not evident from serotyping alone. Sixteen of the 20 most common human *Salmonella* PFGE patterns were identified in *Salmonella* recovered from food animals. The most common human *Salmonella* PFGE pattern, Enteritidis pattern JEGX01.0004 (JEGX01.0003ARS), was associated with more cases of invasive salmonellosis than all other patterns. In food animals, this pattern was almost exclusively (99%) found in *Salmonella* recovered from chickens and was present in poultry meat in every year of the study. Enteritidis pattern JEGX01.0004 (JEGX01.0003ARS) was associated with susceptibility to all antimicrobial agents tested in 94.7% of human and 97.2% of food animal *Salmonella* isolates. In contrast, multidrug resistance (resistance to three or more classes of antimicrobial agents) was observed in five PFGE patterns. Typhimurium patterns JPXX01.0003 (JPXX01.0003 ARS) and JPXX01.0018 (JPXX01.0002 ARS), considered together, were associated with resistance to five or more classes of antimicrobial agents: **a**mpicillin, **c**hloramphenicol, **s**treptomycin, **su**lfonamides and **t**etracycline (ACSSuT), in 92% of human and 80% of food animal *Salmonella* isolates. The information from our study can assist in source attribution, outbreak investigations, and tailoring of interventions to maximize their impact on prevention.

## Introduction

In the United States, non-typhoidal *Salmonella enterica* subsp. *enterica* cause an estimated one million episodes of salmonellosis each year [1] and are the leading cause of hospitalization and death from foodborne illness. The resulting annual economic burden, based on the costs of medical treatment, lost productivity and premature death, is estimated to be in the range of $3.3-4.4 billion [2], [3].

PulseNet is the national molecular surveillance network for foodborne infections and includes in its network the laboratories of state, territorial, and local public health departments, federal food regulatory agencies, veterinary agencies, and agricultural agencies. PulseNet was established by the Centers for Disease Control and Prevention (CDC) and the Association of Public Health Laboratories in 1996 to reduce the time needed to detect, investigate, and control multistate outbreaks caused by foodborne bacterial pathogens. PulseNet laboratories subtype these pathogens using pulsed-field gel electrophoresis (PFGE) and upload the PFGE patterns to a centralized database at CDC [4], [5].

The National Antimicrobial Resistance Monitoring System (NARMS) is a national public health surveillance system that tracks antimicrobial resistance in foodborne bacteria. The NARMS program was established in 1996 as a partnership between the U.S. Food and Drug Administration (FDA), CDC, and the U.S. Department of Agriculture (USDA) and is described on the FDA website [6]. The animal arm of NARMS resides in the USDA-Agricultural Research Service (ARS) laboratory in Athens, GA. In addition to monitoring antimicrobial susceptibility, NARMS partners collaborate on epidemiologic and microbiologic research studies. NARMS also examines foodborne bacteria for genetic relatedness using PFGE, and PFGE patterns are entered into USDA’s VetNet database [7]. The food animal arm of NARMS is described on the USDA web site [8]. PulseNet and VetNet work synergistically to provide information that is important for public health. The PFGE protocols are highly standardized protocols developed by PulseNet to facilitate inter-laboratory comparisons [9].

As part of communicable disease control reporting requirements in Pennsylvania, clinical laboratories routinely submit *Salmonella* isolates to the Pennsylvania Department of Heath Bureau of Laboratories (BOL). At BOL, Salmonella isolates are biochemically identified, serotyped and subtyped by PFGE. USDA’s Food Safety and Inspection Service (FSIS) samples food animals for *Salmonella* during slaughter and processing. *Salmonella* isolates from food animals are tested for susceptibility to antimicrobial agents and then subtyped via PFGE by the USDA-ARS Laboratory in Athens, Georgia.

Our objective was to compare clinical isolates of non-typhoidal *Salmonella* recovered from humans (human *Salmonella*) received as part of routine surveillance at the BOL in Pennsylvania from 2005 through 2011 with *Salmonella* isolates recovered during the same period from food animals (food animal *Salmonella*) at slaughter and processing facilities in the northeastern United States. The most common PFGE patterns observed in human *Salmonella* served as the reference set and included associated invasiveness and antimicrobial susceptibility profiles. Our hypothesis was that subtyping *Salmonella* isolates by PFGE could reveal differences within serotypes in terms of antimicrobial susceptibility patterns and, for human *Salmonella*, differences in food animal sources and invasiveness that were not evident from serotyping alone.

## Materials and Methods

### Sample Sources and Processing

Human and food animal non-typhoidal *Salmonella* isolates received between January 1, 2005 and December 31, 2011, were included in the study. At BOL, human *Salmonella* isolates received as part of the state’s routine operations were grown, identified, and serotyped by the Bacteriology Section, using standard procedures [10], [11]. Food animal *Salmonella* isolates were recovered from carcass rinsates (chickens), carcass swabs (turkeys, cattle, and swine), and ground products (chicken, turkey, and beef) during slaughter and processing at federally inspected facilities in the northeast (Pennsylvania, Maine, Vermont, New Hampshire, New York, Maryland, Connecticut, Rhode Island, Massachusetts, Delaware, New Jersey, Indiana, Ohio, Michigan and Washington, DC) as previously described [12].

PFGE testing of human *Salmonella* isolates was conducted by the BOL according to the CDC-standardized procedure used by all PulseNet-certified laboratories [9]. Gel images were analyzed using BioNumerics software Version 6.6 (Applied Maths, Saint-Martens-Latem, Belgium). All non-typhoidal human *Salmonella* isolates received at the BOL were evaluated by PFGE, except when the same serotype of *Salmonella* was recovered more than once from a patient within a six-month period. In this case, only the first isolate received was tested. PFGE testing of food animal *Salmonella* isolates was done by the animal arm of the NARMS, located in Athens, GA, as previously described [7].

### PFGE Pattern Names

PFGE fingerprints of human *Salmonella* were maintained in a local Pennsylvania database and submitted to CDC’s PulseNet national *Salmonella* database where they were assigned pattern names [4], [5]. The USDA maintains a similar database called USDA-VetNet for PFGE fingerprints of *Salmonella* isolated from food animals [7]. Food animal *Salmonella* pattern names were assigned by USDA-VetNet as previously described [7]. Isolates in the VetNet database were compared to the PulseNet database to capture matching PulseNet pattern names.

### Common Human *Salmonella* Patterns and Shared Common Patterns

The 20 most frequently identified PFGE patterns among human *Salmonella* isolates and with at least two isolates in each year of the study were designated as the most common human *Salmonella* patterns. These 20 patterns included patterns that occurred both sporadically (n = 4,471) and linked to known outbreaks (n = 251). The USDA-VetNet database was then searched for matching patterns in *Salmonella* isolates recovered from food animals during slaughter and processing in northeastern U.S. facilities. The most common human patterns that were also identified in food animal *Salmonella* and are defined here as “shared common patterns.”

### Antimicrobial Susceptibility Testing

Susceptibility to the following classes of antimicrobial agents was tested using the Sensititre semi-automated broth microdilution antimicrobial susceptibility system (Trek Diagnostic Systems Inc., Cleveland, Ohio), with minimum inhibitory concentrations evaluated according to Clinical Laboratory Standards Institute (CLSI) guidelines [13]: (antimicrobial agents in parentheses; resistance breakpoints in brackets): aminoglycosides (amikacin [≥64 µg/mL], gentamicin [≥16 µg/mL], kanamycin [≥64 µg/mL] and streptomycin [≥64 µg/mL]), ß-lactam/ß-lactamase inhibitor combinations (amoxicillin/clavulanic acid [≥32/16 µg/mL]), cephems (cefoxitin [≥32 µg/mL], ceftiofur [≥8 µg/mL] and ceftriaxone [≥4 µg/mL]), penicillins (ampicillin [≥32 µg/mL]), quinolones (ciprofloxacin [≥4 µg/mL]and nalidixic acid [≥32 µg/mL]), folate pathway inhibitors (sulfisoxazole [≥512 µg/mL], trimethoprim/sulfamethoxazole [≥4/76 µg/mL]), phenicols (chloramphenicol [≥32 µg/mL]), and tetracyclines (tetracycline [≥16 µg/mL]). For antimicrobial agents without CLSI approved standards, NARMS interpretive criteria as established by the NARMS working group were used and quality control strains were as previously described [8]. Multidrug resistance was defined as resistance to three or more classes of antimicrobial agents. Of the 4,235 human *Salmonella* isolates with common shared patterns, 467 (11%) were tested for susceptibility to antimicrobial agents. Limited resources precluded testing all of the isolates. The isolates chosen for susceptibility testing included 267 randomly selected isolates originating from Pennsylvania that were tested by the human arm of NARMS as part of its routine surveillance program [14]. The Pennsylvania Department of Agriculture tested an additional 200 human *Salmonella* isolates sampled from the 16 shared common patterns. NARMS tested all 275 of the food animal *Salmonella* isolates having shared common patterns for antimicrobial susceptibility as previously described [12].

### Correlation of Invasiveness with PFGE Patterns

Isolation of *Salmonella* from human blood was used as an indicator of invasive disease [15]. The statistical association between each PFGE pattern and invasiveness was tested via a 2×2 contingency table and evaluated on the basis of the conditional maximum likelihood estimate of Odds Ratio (OR) and the mid-p test (two-tailed p value) [16]. PFGE patterns with OR≥1 and p≤0.05 were interpreted as associated with increased invasiveness; patterns with OR≤1 and p≤0.05 were interpreted as associated with decreased invasiveness.

### Correlation of Antimicrobial Resistance with Invasiveness

A two-tailed p value obtained with a Fisher’s exact test (GraphPad QuickCalcs, GraphPad Software, Inc., San Diego, CA) was used to evaluate the relationship between antimicrobial resistance and invasiveness.

Regarding data for *Salmonella* recovered from animals: ACUC approval was not needed as the isolates were obtained as part of the National Antimicrobial Resistance Monitoring System (NARMS) from the USDA Food Safety and Inspection Service (FSIS) as part of their Salmonella PR/HACCP verification testing program (http://www.ars.usda.gov/Main/docs.htm?docid=6750&page=2). Accessed 2013 September 19.

## Results

### PFGE Patterns Found both in Human and Food Animal *Salmonella* Isolates

A total of 11,967 human *Salmonella* and 2,187 food animal *Salmonella* isolates were submitted for laboratory testing during the study period (2005–2011). The food animal *Salmonella* isolates were recovered from a total of 65,655 animals tested for *Salmonella* during slaughter and processing at federally inspected facilities in the northeastern U.S., and represented an overall yield of 3.3% (Table 1). The 65,655 animals tested for *Salmonella* included 42,368 (64.5%) cattle, 10,412 (15.9%) swine, 9,661(14.7%) chickens and 3,214 (4.9%) turkeys. *Salmonella* was isolated from a total of 2,187 samples (Table 1). Of the 2,187 *Salmonella-*positive samples, 1,194 (54.6%) were recovered from chickens, 472 (21.6%) from cattle, 282 (12.9%) from turkeys and 239 (10.9%) from swine. A total of 2,083 of the 2,187 food animal *Salmonella* isolates were available to VetNet for PFGE, antimicrobial susceptibility testing and comparison with human *Salmonella* isolates.

**Table 1:** Food Animal Sources of *Salmonella* Recovered at Federally Inspected Slaughter and Processing Facilities in the Northeastern U.S.^1^ with Human Isolates Shown for Reference.

Year	No. Chickens Tested	No. (%) Chickens Positive for *Salmonella*	No. Cattle Tested	No. (%) Cattle Positive for *Salmonella*	No. Turkeys Tested	No. (%)TurkeysPositive forSalmonella	No. Swine Tested	No. (%) Swine Positive for *Salmonella*	Total No. Samples Inspected	Total No. (%) Positive for *Salmonella*	No. Human Salmonella Received	No. 20 Most Common Human *Salmonella*
2005	1714	315 (18.4)	9,754	102 (1.0)	175	39 (22.3)	2,191	61 (2.8)	13,834	517 (3.7)	1,537	549
2006	1,947	271 (13.9)	8,449	117 (1.4)	730	74 (10.1)	2,346	78 (3.3)	13,472	540 (4.0)	1,686	749
2007	1,684	172 (10.2)	6,539	76 (1.2)	709	49 (6.9)	1,643	27 (1.6)	10,575	324 (3.1)	1,829	706
2008	1,002	103 (10.3)	6,241	90 (1.4)	276	43 (15.6)	1,342	26 (1.9)	8,861	262 (3.0)	1,680	645
2009	989	95 (9.6)	4,005	19 (0.5)	376	29 (7.7)	1,099	13 (1.2)	6,469	156 (2.4)	1,706	662
2010	1,254	146 (11.6)	3,099	29 (0.9)	573	38 (6.6)	1,137	24 (2.1)	6,063	237 (3.9)	1,749	716
2011	1,071	92 (8.6)	4,281	39 (0.9)	375	10 (2.7)	654	10 (1.5)	6,381	151 (2.4)	1,778	695
TOTAL	9,661	1,194 (12.4)	42,368	472 (1.1)	3,214	282 (8.8)	10,412	239 (2.3)	65,655	2,187 (3.3)	11,967	4722

1 Pennsylvania, Maine, Vermont, New Hampshire, New York, Maryland, Connecticut, Rhode Island, Massachusetts, Delaware, New Jersey, Indiana, Ohio, Michigan and Washington, DC.

### Common Human *Salmonella* Patterns

**Figure 1:**
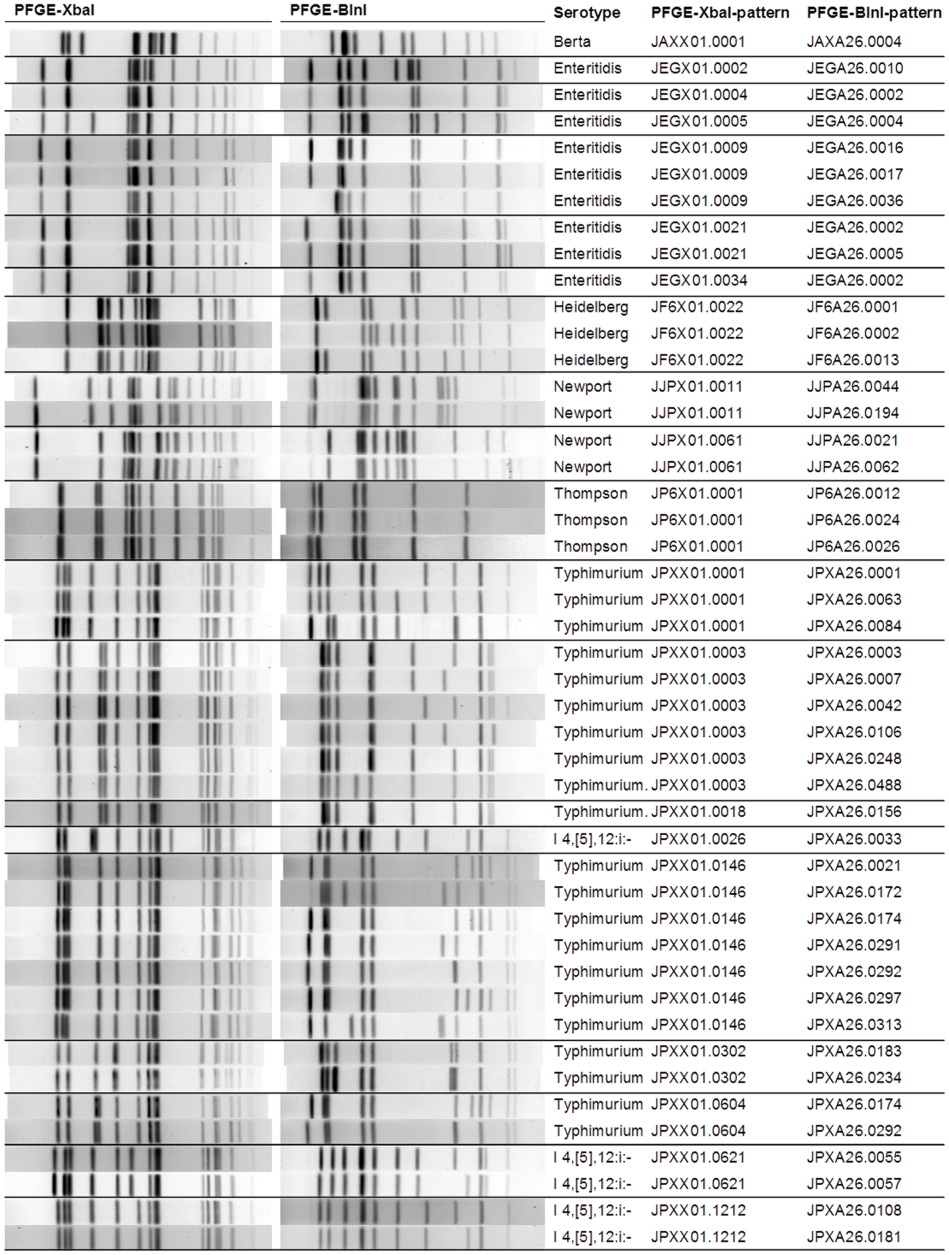
DNA fingerprints of the 20 most common human *Salmonella* patterns. PFGE was conducted as described in Materials and Methods, with restriction endonucleases XbaI and BlnI. BlnI patterns represented at least three times in the dataset are shown.

The 20 most common human *Salmonella* XbaI patterns are shown in Fig. 1 and described in Table 2. These patterns represent 39% of the human *Salmonella* isolates (4,722/11,967) from Pennsylvania described in this paper. Six serotypes–Berta, Enteritidis, Heidelberg, Newport, Thompson, and Typhimurium (including variant 5-)–and one antigenic formula (I 4,[5],12:i:-) were represented among these 20 patterns. Each XbaI pattern is shown with all associated BlnI patterns that occurred at least three times in the dataset. One to seven different BlnI patterns were associated with a particular XbaI pattern.

**Table 2:** Pennsylvania’s Most Common Human *Salmonella* PFGE Patterns Also Found in *Salmonella* Recovered from Food Animals Processed in the Northeastern U.S.

Serotype	PulseNet XbaI Pattern^1^	No. (% of All Human *Salmonella*)^2^	No.Linked toOutbreaks	No. from Blood	% from Blood within Pattern^3^	Association ofPFGE Pattern withBlood^3^ Odds Ratio(95% CI); p	CorrespondingVetNet Pattern^4^		No. (%)^5^of FoodAnimal*Salmonella*	No.fromCattle	No.fromChicken	No.fromSwine	No.fromTurkey
Berta	JAXX01.0001	49 (0.41)	0	5	10.20	2.00 (0.70-4.75); 0.17	JAXX01.0003 ARS		3 (0.14)	0	0	0	3
Enteritidis	JEGX01.0002	235 (1.96)	3	14	5.96	1.11 (0.62-1.87); 0.68	JEGX01.0017 ARS		0	0	0	0	0
Enteritidis	JEGX01.0004	1,968 (16.45)	79	130	6.61	1.30 (1.06-1.58); 0.01	JEGX01.0003 ARS		106 (5.09)	1	105	0	0
Enteritidis	JEGX01.0005	426 (3.56)	12	35	8.22	1.60 (1.11-2.26); 0.01	JEGX01.0002 ARS		65 (3.12)	1	64	0	0
Enteritidis	JEGX01.0009	144 (1.20)	1	15	10.42	2.06 (1.16-3.46); 0.02	JEGX01.0022 ARS		0	0	0	0	0
Enteritidis	JEGX01.0021	331 (2.77)	24	21	6.34	1.19 (0.74-1.84); 0.43	JEGX01.0010 ARS; JEGX01.0052 ARS^6^		1 (0.05)	0	1	0	0
Enteritidis	JEGX01.0034	176 (1.47)	11	1	0.57	0.10 (0.00-0.49); 0.00	JEGX01.0005 ARS		14 (0.67)	0	14	0	0
Heidelberg	JF6X01.0022	151 (1.26)	18	16	10.60	2.10 (1.21-3.48); 0.01	JF6X01.0015 ARS		30 (1.44)	3	25	0	2
Newport	JJPX01.0011	54 (0.45)	0	0	0	0.00 (0.00-1.00); 0.05	JJPX01.0204 ARS		1 (0.05)	0	1	0	0
Newport	JJPX01.0061	72 (0.60)	28	0	0	0.00 (0.00-0.74); 0.02	JJPX01.0069 ARS		1 (0.05)	0	0	1	0
Thompson	JP6X01.0001	54 (0.45)	0	3	5.56	1.03 (0.25-2.95); 0.90	JP6X01.0024 ARS		1 (0.05)	0	1	0	0
Typhimurium	JPXX01.0001	53 (0.44)	2	3	5.66	1.05 (0.26-3.02); 0.87	JPXX01.0021 ARS		1 (0.05)	0	1	0	0
Typhimurium	JPXX01.0003	113 (0.94)	2	9	7.96	1.52 (0.72-2.91); 0.24	JPXX01.0003 ARS		18 (0.86)	6	5	3	4
Typhimurium	JPXX01.0018	81 (0.68)	0	2	2.47	0.44 (0.07-1.51); 0.24	JPXX01.0002 ARS		2 (0.10)	1	0	1	0
Typhimurium	JPXX01.0146	278 (2.32)	16	1	0.36	0.06 (0.00-0.31); 0.00	JPXX01.0081 ARS		5 (0.24)	0	1	4	0
Typhimurium	JPXX01.0302	149 (1.25)	46	3	2.01	0.36 (0.09-0.99); 0.05	JPXX01.0106 ARS		2 (0.10)	1	1	0	0
Typhimurium	JPXX01.0604	61 (0.51)	3	1	1.64	0.29 (0.01-1.49); 0.18	JPXX01.0079 ARS		10 (0.48)	1	7	0	2
Typhimurium;I 4,[5],12:i:-	JPXX01.0026	61 (0.51)	4	1	1.64	0.29 (0.01-1.49); 0.18	JPXX01.0308 ARS; TERX01.0010 ARS		0	0	0	0	0
I 4,[5],12:i:-	JPXX01.0621	219 (1.83)	2	8	3.65	0.66 (0.30-1.28); 0.25	TERX01.0001 ARS		15 (0.72)	2	12	1	0
Typhimurium;I 4,[5],12:i:-	JPXX01.1212	47 (0.39)	0	3	6.38	1.20 (0.29-3.45); 0.72	JPXX01.0304 ARS; TERX01.0008 ARS		0	0	0	0	0
TOTAL		4,722 (39.46)	251	271	NA		TOTAL	275 (13.20)		16	238	10	11

1 Pattern names include a three-letter code that indicates serotype (e.g., JEG for serotype Enteritidis in the pattern names JEGX01.0004 and JEGA26.0002), followed by a three-character code for.

restriction enzyme (e.g., X01 for XbaI in JEGX01.0004; A26 for BlnI in JEGA26.0002), and a four-digit number for the unique strain designation (e.g., 0004 in JEGX01.0004) (Gerner-Smidt et al., 2006).

2 Percentage of total number of human *Salmonella* isolates (11,967).

3 Number of human *Salmonella* isolates recovered from blood for a particular pattern expressed as a percentage of the total number from all sources for that PFGE pattern; e.g., for Berta XbaI.

pattern JAXX01.0001, (5/49)×100 = 10.20%. Statistical method for determining Odds Ratio is described in Materials and Methods.

4 VetNet’s pattern nomenclature conforms to PulseNet’s nomenclature, with three exceptions: (1) PulseNet uses a single three-letter code (JPX) to designate both serotype Typhimurium and antigenic formula I 4,[5],12:i:- whereas VetNet uses two different codes, JPX for Typhimurium and TER for I 4,[5],12:i:-; (2) the four-digit strain designation codes are different in the two systems; and (3) “ARS” (for Agricultural Research Service of the USDA) is appended to VetNet pattern names.

5 Percentage of the total number of animal *Salmonella* isolates (2,083).

6 VetNet identified two different patterns within PulseNet pattern JEGX01.0021.

Sixteen (80%) of the 20 most common human *Salmonella* patterns shown in Fig. 1 were also found among the 2,083 food animal *Salmonella* isolates (Table 2). A total of 4,235 (35%) of the 11,967 human *Salmonella* isolates shared these 16 PFGE patterns with 275 (13%) of the 2,083 food animal *Salmonella* isolates. All six serotypes and the antigenic formula identified in the 20 most common human *Salmonella* patterns were also represented among the shared common patterns.

The most frequently observed shared common pattern from human *Salmonella* isolates was Enteritidis pattern JEGX01.0004 (JEGX01.0003ARS), which accounted for 16% of all human *Salmonella*. Among food animal *Salmonella*, this pattern was the second most common pattern, accounting for 5% of all food animal *Salmonella* isolates. Serotype Kentucky pattern JGPX01.0027 (JGPX01.0220 ARS) was the most common PFGE pattern in food animal *Salmonella*, occurring in 13% of food animal *Salmonella* isolates (data not shown). This pattern was rarely seen in BOL human *Salmonella* isolates (0.25%).

Of the 275 food animal *Salmonella* isolates with shared common patterns, 238 (87%) were recovered from chicken, 16 (6%) from cattle, 11 (4%) from turkey and 10 (4%) from swine (Table 2). Sixty seven percent (n = 186) of these 275 food animal *Salmonella* isolates belonged to serotype Enteritidis, and all but two isolates were recovered from chicken. The most common Enteritidis pattern in *Salmonella* from chickens was JEGX01.0004 (JEGX01.0003ARS) (n = 105/184; 57%). Chicken was also the most common source of Heidelberg pattern JF6X01.0022 (JF6X01.0015 ARS), with 25 of 30 isolates (83%) recovered from chicken (Table 2).

In contrast to the strong association of S. Enteritidis and Heidelberg patterns with chicken, Typhimurium pattern JPXX01.0003 (JPXX01.0003 ARS) (n = 18) was observed in *Salmonella* recovered in comparable numbers from cattle (n = 6), chicken (n = 5), turkey (n = 4) and swine (n = 3). The second most common food animal Typhimurium pattern, JPXX01.0604 (JPXX01.0079 ARS) (n = 10) was primarily recovered from chicken (n = 7). The two isolates of pattern JPXX01.0018 (JPXX01.0002 ARS) were recovered from cattle and swine, respectively, and Typhimurium pattern JPXX01.0146 (JPXX01.0081 ARS) (n = 5) was recovered primarily from swine (n = 4). Berta pattern JAXX01.0001 (JAXX01.0003 ARS) (n = 3) was found exclusively in turkey.

All 16 shared common patterns were found in human *Salmonella* during each of the seven years of the study. Three of the 16 shared common patterns, Enteritidis patterns JEGX01.0004 (JEGX01.0003 ARS), JEGX01.0005 (JEGX01.0002 ARS) and JEGX01.0034 (JEGX01.0005 ARS), were also found in food animal *Salmonella* during each of the seven years of the study. Similarly, Heidelberg pattern JF6X01.0022 (JF6X01.0015 ARS) was recovered from food animals in each of five years, and Typhimurium pattern JPXX01.0003 (JPXX01.0003 ARS) in each of four years (data not shown).

### Unshared PFGE Patterns

A total of 261 different patterns were identified among the 11,967 human *Salmonella* isolates in Pennsylvania, and 794 were identified among the 2,083 food animal *Salmonella* isolates (data not shown). Many of the identified human patterns were not shared by food animal *Salmonella* isolates, and many of the identified food animal *Salmonella* patterns were not shared by human *Salmonella* isolates. Of the 2,083 food animal *Salmonella* isolates, only 1,034 (50%) shared PFGE patterns with the human *Salmonella* patterns that had been assigned pattern names. Of the 11,967 human *Salmonella* isolates, only 5,266 (44%) had named patterns found among the 2,083 food animal *Salmonella* isolates recovered from northeastern slaughter and processing facilities.

Of the 20 most common human *Salmonella* patterns (Fig. 1 and Table 2), two Enteritidis patterns (JEGX01.0002 and JEGX01.0009) and two Typhimurium/I 4,[5],12:i:- patterns (JPXX01.0026 and JPXX01.1212) were not observed among food animal *Salmonella* isolates. Pattern JEGX01.0002 was found to be associated with travel. Travel histories were available for 200 persons associated with *Salmonella* pattern JEGX01.0002; of these, 164 (82%) reported traveling outside the U.S., notably to the Dominican Republic (n = 71) and Mexico (n = 53).

### Association of Invasiveness with PFGE Patterns

Isolation from blood was used as an indicator of invasiveness. A total of 646 *Salmonella* isolates from humans were recovered from blood, representing 5.4% of the 11,967 human *Salmonella* isolates tested (Table 2). Four patterns were found to be significantly associated with increased frequency of isolation from blood: Enteritidis JEGX01.0004 (JEGX01.0003 ARS) (OR = 1.30; p = 0.01), Enteritidis JEGX01.0005 (JEGX01.0002 ARS) (OR = 1.60; p = 0.014), Enteritidis JEGX01.0009 (JEGX01.0022 ARS) (OR = 2.06; p = 0.02) and Heidelberg JF6X01.002 (JF6X01.0015 ARS) (OR = 2.10; p = 0.01). Five patterns were found to be significantly associated with decreased frequency of isolation from blood: Enteritidis JEGX01.0034 (JEGX01.0005 ARS) (OR = 0.10; p = 0.00), Newport patterns JJPX01.0011 (JJPX01.0204 ARS) (OR = 0.00; p = 0.05) and JJPX01.0061 (JJPX01.0069 ARS) (OR = 0.00; p = 0.02), and Typhimurium patterns JPXX01.0146 (JPXX01.0081 ARS) (OR = 0.06; p = 0.00) and JPXX01.0302 (JPXX01.0106 ARS) (OR = 0.36; p = 0.05).

### Antimicrobial Susceptibility Associated with Shared Common Patterns

Of the 4,235 human *Salmonella* isolates with shared common patterns, 467 (11%) were tested for susceptibility to antimicrobial agents (Table 3). Of these 467, a total of 367 (79%) were pansusceptible, 100 (21%) were resistant to at least one class of antimicrobial agent, 75 (16%) were MDR, and 62 (13%) were resistant to five or more classes of antimicrobial agents. Of the 275 food animal *Salmonella* isolates, 226 (82%) were pansusceptible, 49 (18%) were resistant to at least one class of antimicrobial agents, 40 (14%) were MDR, and 16 (6%) were resistant to five or more classes of antimicrobial agents.

**Table 3:** Antimicrobial resistance associated with the 16 most common human *Salmonella* PFGE patterns shared by food animal *Salmonella*.

Human *Salmonella*									Food Animal *Salmonella*							
Serotype^1^	PulseNetXbaI Pattern	No.Tested	No. (%)Pansusceptible^7^	No. (%)^5^Resistant to1-2AntimicrobialClasses^7^	No. (%)^5^MDR^2^	No. (%)^5^Resistantto ≥5AntimicrobialClasses^7^	No.Resist-antto Nal^3^	No.Resist-antto Axo^3^	CorrespondingVetNet-XbaI-pattern	No.Tested	No. (%)Pansusceptible^7^	No. (%)^6^Resistant to1-2AntimicrobialClasses^7^	No. (%)^6^MDR^2^	No. (%)^6^Resistantto ≥5AntimicrobialClasses^7^	No.Resist-antto Nal^3^	No.Resist-antto Axo^3^
Berta	JAXX01.0001	16	9 (56.3)	2 (12.5)	5 (31.3)	1 (6.3)	1	2	JAXX01.0003 ARS	3	0 (0.0)	0 (0.0)	3 (100.0)	0 (0.0)	0	1
Enteritidis	JEGX01.0004	114	108 (94.7)	5 (4.4)	1 (0.9)	0 (0.0)	1	0	JEGX01.0003 ARS	106	103 (97.2)	3 (2.8)	0 (0.0)	0 (0.0)	0	0
Enteritidis	JEGX01.0005	25	25 (100.0)	0 (0.0)	0 (0.0)	0 (0.0)	0	0	JEGX01.0002 ARS	65	65 (100.0)	0 (0.0)	0 (0.0)	0 (0.0)	0	0
Enteritidis	JEGX01.0021	33	27 (81.8)	6 (7.3)	0 (0.0)	0 (0.0)	0	0	JEGX01.0010 ARS JEGX01.0052 ARS^4^	1	0 (0.0)	1 (100.0)	0 (0.0)	0 (0.0)	0	0
Enteritidis	JEGX01.0034	22	21 (95.5)	1 (4.5)	0 (0.0)	0 (0.0)	0	0	JEGX01.0005 ARS	14	12 (85.7)	2 (14.3)	0 (0.0)	0 (0.0)	0	0
Heidelberg	JF6X01.0022	14	13 (92.9)	0 (0.0)	1 (7.1)	0 (00.)	0	1	JF6X01.0015 ARS	30	18 (60.0)	2 (6.7)	10 (33.3)	0 (0.0)	0	10
Newport	JJPX01.0011	9	9 (100.0)	0 (0.0)	0 (0.0)	0 (0.0)	0	0	JJPX01.0204 ARS	1	1 (100.0)	0 (0.0)	0 (0.0)	0 (0.0)	0	0
Newport	JJPX01.0061	13	13 (100.0)	0 (0.0)	0 (0.0)	0 (00.)	0	0	JJPX01.0069 ARS	1	1 (100.0)	0 (0.0)	0 (0.0)	0 (0.0)	0	0
Thompson	JP6X01.0001	8	8 (100)	0 (0.0)	0 (0.0)	0 (0.0)	0	0	JP6X01.0024 ARS	1	1 (100.0)	0 (0.0)	0 (0.0)	0 (0.0)	0	0
Typhimurium	JPXX01.0001	13	10 (76.9)	1 (7.7)	2 (15.4)	1 (7.7)	0	1	JPXX01.0021 ARS	1	1 (100.0)	0 (0.0)	0 (0.0)	0 (0.0)	0	0
Typhimurium	JPXX01.0003	38	0 (0.0)	1 (2.6)	37 (97.4)	37 (97.4)	0	0	JPXX01.0003 ARS	18	0 (0.0)	1 (5.6)	17 (94.4)	14 (78.0)	0	0
Typhimurium	JPXX01.0018	27	0 (0.0)	2 (7.4)	25 (92.6)	23 (85.2)	0	0	JPXX01.0002 ARS	2	0 (0.0)	0 (0.0)	2 (100.0)	2 (100.0)	0	0
Typhimurium	JPXX01.0146	24	23 (95.8)	1 (4.2)	0 (0.0)	0 (0.0)	0	0	JPXX01.0081 ARS	5	5 (100.0)	0 (0.0)	0 (0.0)	0 (0.0)	0	0
Typhimurium	JPXX01.0302	22	20 (90.9)	1 (4.5)	1 (4.5)	0 (0.0)	1	0	JPXX01.0106 ARS	2	2 (100.0)	0 (0.0)	0 (0.0)	0 (0.0)	0	0
Typhimurium	JPXX01.0604	13	13 (100.0)	0 (0.0)	0 (0.0)	0 (0.0)	0	0	JPXX01.0079 ARS	10	10 (100.0)	0 (0.0)	0 (0.0)	0 (0.0)	0	0
I 4,[5],12:i:-	JPXX01.0621	76	68 (89.5)	5 (6.6)	3 (3.9)	0 (0.0)	0	1	TERX01.0001 ARS	15	7 (46.7)	0 (0.0)	8 (53.3)	0 (0.0)	0	7
TOTAL		467	367 (78.6)	25 (5.4)	75 (16.1)	62 (13.3)	3	5	TOTAL	275	226 (82.2)	9 (3.3)	40 (14.5)	16 (5.8)	0	18

1 Typhimurium includes var. O5-.

2 Resistant to at least three classes of antimicrobial agents.

3 Abbreviations: nalidixic acid (Nal); ceftriaxone (Axo).

4 VetNet identified two different patterns within PulseNet pattern JEGX01.0021.

5 Based on 467 isolates tested for antimicrobial susceptibility.

6 Based on 275 isolates tested for antimicrobial susceptibility.

7 Antimicrobial classes (antimicrobial agents in parentheses; resistance breakpoints in brackets): aminoglycosides (amikacin [≥64 µg/mL], gentamicin [≥16 µg/mL], kanamycin [≥64 µg/mL] and streptomycin [≥64 µg/mL]), ß-lactam/ß-lactamase inhibitor combinations (amoxicillin/clavulanic acid [≥32/16 µg/mL]), cephems (cefoxitin [≥32 µg/mL], ceftiofur [≥8 µg/mL] and ceftriaxone [≥4 µg/mL]), penicillins (ampicillin [≥32 µg/mL]), quinolones (ciprofloxacin [≥4 µg/mL]and nalidixic acid [≥32 µg/mL]), folate pathway inhibitors (sulfisoxazole [≥512 µg/mL], trimethoprim/sulfamethoxazole [≥4/76 µg/mL]), phenicols (chloramphenicol [≥32 µg/mL]), and tetracyclines (tetracycline [≥16 µg/mL]).

Multidrug resistance (resistance to ≥3 classes of antimicrobial agents) was associated with eight of the 16 shared common patterns in human *Salmonella* (Table 3). Five of these patterns were also associated with multidrug resistance in food animal *Salmonella*: Berta pattern JAXX01.0001 (JAXX01.0003 ARS), Heidelberg pattern JF6X01.0022 (JF6X01.0015 ARS), Typhimurium patterns JPXX01.0003 (JPXX01.0003 ARS) and JPXX01.0018 (JPXX01.0002 ARS), and I 4,[5],12:i:- pattern JPXX01.0621 (TERX01.0001 ARS). The incidence of multidrug resistance in both human and food animal *Salmonella* isolates exceeded 90% for Typhimurium patterns JPXX01.0003 (JPXX01.0003 ARS) and JPXX01.0018 (JPXX01.0002 ARS) (Table 3), patterns that differ by two bands (Fig. 1). When these two patterns were considered together, a total of 58 (89%) of the 65 human and 15 (75%) of the 20 food animal Typhimurium isolates were resistant to the following five antimicrobial agents: ampicillin, chloramphenicol, streptomycin, sulfisoxazole, and tetracycline (the ACSSuT phenotype and resistance to five classes of antimicrobial agents). Two of the human *Salmonella* isolates exhibited resistance to amoxicillin/clavulanic acid and ceftiofur in addition to the ACSSuT phenotype. The food animal *Salmonella* isolates exhibiting the ACSSuT phenotype were recovered from cattle (n = 5), chickens (n = 4), swine (n = 3) and turkeys (n = 3). An additional isolate recovered from a turkey exhibited the ACSSuT phenotype plus resistance to gentamicin, and three additional isolates (two from cattle; one from swine) lacked only the streptomycin resistance of this phenotype (ACSuT). One human isolate and three food animal isolates with patterns JPXX01.0003 (JPXX01.0003 ARS) and JPXX01.0018 (JPXX01.0002 ARS) were resistant only to streptomycin and sulfisoxazole. Collectively Typhimurium patterns JPXX01.0003 (JPXX01.0003 ARS) and JPXX01.0018 (JPXX01.0002 ARS) were associated with resistance to five or more classes of antimicrobial agents in 60 (92%) of the 65 human *Salmonella* and 16 (80%) of the 20 food animal *Salmonella*.

Of the 15 food animal *Salmonella* isolates exhibiting I 4,[5],12:i:- pattern JPXX01.0621 (TERX01.0001 ARS), seven were resistant to amoxicillin/clavulanic acid, ampicillin, cefoxitin, ceftiofur and ceftriaxone. Of the 76 susceptibility-tested human *Salmonella* isolates exhibiting patternJPXX01.0621 (TERX01.0001 ARS), only one exhibited the exact resistance pattern. A second human *Salmonella* isolate exhibited a closely related resistance profile: the isolate was intermediate instead of resistant to ceftriaxone. The resistance patterns of the additional MDR food animal *Salmonella* isolates and human *Salmonella* isolate with pattern JPXX01.0621 (TERX01.0001 ARS) did not match.

Although we did not find resistance to the fluoroquinolone ciprofloxacin in any human or food animal *Salmonella* isolate, three human *Salmonella* isolates, Berta pattern JAXX01.0001, Enteritidis pattern JEGX01.0004 (JEGX01.0003 ARS) and Typhimurium pattern JPXX01.0302 (JPXX01.0106 ARS), were resistant to nalidixic acid (Nal), an indicator of decreasing susceptibility to quinolones [14].

### Invasiveness and Antimicrobial Resistance to Antimicrobial Agents

Of the 467 human *Salmonella* isolates tested for antimicrobial susceptibility, 367 were pansusceptible, and 100 were resistant to one or more antimicrobial agents (Table 3). Twelve (3.2%) of the 367 pansusceptible isolates and 10 (10%) of the 100 isolates resistant to one or more antimicrobial agents were recovered from blood. This difference was found to be significant (p = 0.0129). Included among the 10 resistant blood isolates were four Typhimurium pattern JPXX01.0003 (JPXX01.0003 ARS) isolates, two Enteritidis pattern JEGX01.0021 (JEGX01.0010 ARS and JEGX01.0052 ARS), one each of Typhimurium patterns JPXX01.0018 (JPXX01.0002 ARS) and JPXX01.0146 (JPXX01.0081 ARS), one Berta pattern JAXX01.0001 (JAXX01.0003 ARS ), and one nalidixic acid-resistant Enteritidis pattern JEGX01.0004 (JEGX01.0003 ARS).

## Discussion

We compared clinical isolates of *Salmonella* recovered from ill humans received as part of routine operations in Pennsylvania during 2005-2011 with *Salmonella* isolates collected during the same period from food animals at federally inspected slaughter and processing facilities in the northeastern U.S. We also correlated PFGE patterns of *Salmonella* from humans with animal sources, invasiveness, and antimicrobial susceptibility profiles. Our analysis was based on the most common human *Salmonella* PFGE patterns identified during this period, regardless of serotype. This approach differs from many studies because of its focus on PFGE patterns rather than serotypes and on PFGE patterns that are frequently observed with sporadic disease, rather than solely on patterns linked to outbreaks. However, it is important to note that outbreak isolates were a portion of the overall number of isolates in this study. This is the first study to compare the PFGE patterns of human *Salmonella* isolates recovered during routine surveillance with food animal *Salmonella* isolates recovered at federally inspected slaughter and processing facilities. We found that subtyping *Salmonella* isolates by PFGE revealed differences within serotypes in terms of antimicrobial susceptibility patterns and, for human *Salmonella*, differences in food animal sources and invasiveness that were not evident from serotyping alone.

We found that most (16) but not all of the 20 most common PFGE patterns identified in human *Salmonella* in Pennsylvania were also found in *Salmonella* recovered from food animals in the northeast. The fact that some patterns within a particular serotype were observed in human *Salmonella* but not food animal *Salmonella* and vice versa demonstrates the utility of subtyping isolates within a serotype. Others have previously demonstrated PFGE pattern diversity within a serotype [17]-[20], but such diversity is a critical observation that is often overlooked. PFGE pattern diversity may explain why morbidity and mortality vary within a serotype and could be useful in assessing the effectiveness of control measures.

Further, the absence of some of the most common human *Salmonella* PFGE patterns from food animal *Salmonella* isolates suggests that sources other than food animals play a role in clinical illness: e.g., fresh produce, nuts and other foods [21], [22], suggesting that there is a vast biodiversity associated with food production that is often overlooked.

Although chickens represented only 14.7% of the 65,655 food animals tested, they represented 54.6% of the 2,187 food animals that were positive for *Salmonella* and were the source of 87% of the isolates with shared common patterns. Chicken consumption has been recognized as one of the risk factors for developing salmonellosis caused by serotypes Enteritidis [23]-[25] and Heidelberg [26], and our PFGE results support these associations. Enteritidis pattern JEGX01.0004 (JEGX01.0003 ARS) was the most common human *Salmonella* pattern in Pennsylvania, accounting for 1,968 (16%) of the 11,967 human Salmonella isolates and 130 (20%) of the 646 invasive salmonellosis cases. The presence of *Salmonella* Enteritidis pattern JEGX01.0004 (JEGX01.0003) in chickens during every year of this study suggests the potential for a significant impact on public health and requires further investigation to determine what attributes sustain the persistence of this strain and what measures could reduce its incidence. This further supports our assertion that consideration of strain diversity (i.e., PFGE pattern diversity) is a critical factor for developing control measures.

The frequent occurrence of human salmonellosis associated with pattern JEGX01.0004 (JEGX01.0003 ARS) makes case investigations difficult unless there is a strong epidemiological connection linking the cases. Lacking such a connection, salmonellosis cases associated with this pattern are generally given less scrutiny by epidemiologists, in spite of the fact that the number of cases associated with this pattern in Pennsylvania (n = 1,968) is much greater than the number of identified outbreak-associated isolates for all patterns combined (n = 251) (Table 2). S. Enteritidis pattern JEGX01.0004 (JEGX01.0003 ARS) was associated with a national outbreak involving approximately 1,939 illnesses [27] and recalls of more than 500 million shell eggs [28]. New regulations governing egg safety in the U.S. [29] may help to reduce the incidence of salmonellosis associated with pattern JEGX01.0004 (JEGX01.0003 ARS). However, since our study has demonstrated seven years of this pattern’s high level of persistence in poultry meat, it is reasonable to conclude that *Salmonella* Enteritidis pattern JEGX01.0004 (JEGX01.0003 ARS) is persistent and endemic in poultry. Efforts focused on reducing *Salmonella* in broiler flocks have met with very encouraging results in Denmark [30], [31] and the United Kingdom [32]. Data collected in the coming years will provide critical information regarding the potential reduction of *Salmonella* Enteritidis pattern JEGX01.0004 (JEGX01.0003 ARS) in the United States.

Eleven of the 16 shared PFGE patterns were associated with susceptibility to all antimicrobial agents tested or resistance to fewer than three classes of antimicrobial agents in food animal *Salmonella* (Table 3). This is encouraging from an antimicrobial resistance perspective, indicating that not all PFGE subtypes and serotypes acquire the same degree or type of resistance. Multidrug resistance was associated with Berta pattern JAXX01.0001 (JAXX01.0003 ARS), Heidelberg pattern JF6X01.0022 (JF6X01.0015 ARS), Typhimurium patterns JPXX01.0003 (JPXX01.0003 ARS) and JPXX01.0018 (JPXX01.0002 ARS), and I 4,[5],12:i:- pattern JPXX01.0621 (TERX01.0001 ARS) in both human and food animal *Salmonella*. The high degree of correspondence between PFGE patterns and antimicrobial resistance profiles for Typhimurium patterns JPXX01.0003 (JPXX01.0003 ARS) and JPXX01.0018 (JPXX01.0002 ARS) in human and food animal *Salmonella* isolates contributes to the existing body of evidence linking contaminated food and human salmonellosis. Supporting this correlation is the fact that ACSSuT-associated *Salmonella* pattern JPXX01.0003 (JPXX01.0003 ARS) was linked to a 2003 outbreak of *Salmonella-*contaminated ground beef [33]. The broad representation of MDR patterns JPXX01.0003 (JPXX01.0003 ARS) and JPXX01.0018 (JPXX01.0002 ARS) in all four types of food animals contrasts sharply with the strong association of most other patterns with chicken (Table 2), although this may be confounded by differences in the number of animals of each type that were tested (Table 1). Monitoring the incidence of these patterns and their associated resistance phenotypes could aid in assessing the effect of efforts to reduce the use of antimicrobial agents for growth promotion and disease prevention in food animals on public health.

Differences in the percentage of blood isolates representing each pattern suggest that there may be differences in invasiveness among *Salmonella* having different patterns, even for patterns within a serotype. Further work is needed to determine why certain patterns were associated with increased or decreased invasiveness.

In our sample of 467 human *Salmonella* isolates that were tested for susceptibility to antimicrobial agents, we did not observe a direct correlation between resistance and invasiveness within specific PFGE patterns; however, we did find that the percentage of antimicrobial-resistant isolates that were recovered from blood (10%) was approximately three times the percentage found to be pansusceptible (3.2%; p = 0.0129). Further work is needed to confirm this correlation.

For some of the four unshared common patterns, sources other than food animals slaughtered or processed in northeastern U.S. facilities are likely. For example, we found that S. Enteritidis pattern JEGX01.0002 was associated with travel outside the U.S., especially to the Dominican Republic and Mexico. Another common human *Salmonella* pattern that was not noted in food animal *Salmonella* isolates, Enteritidis pattern JEGX01.0009, has been associated with imported food: *Salmonella* with this pattern has been recovered from shrimp imported from Bangladesh and pompano from Taiwan (PulseNet national PulseNet *Salmonella* database was searched on 2012-06-11 and included data uploaded between 2008-06-12 and 2012-06-11).

In some cases, the absence of common human *Salmonella* PFGE patterns in food animal *Salmonella* may have resulted from testing an insufficient number of animals. From 2005 through 2011, the number of food animals tested for *Salmonella* in northeastern slaughter and processing facilities declined by more than half–from 13,834 in 2005 to 6,381 in 2011. Although Typhimurium pattern JPXX01.1212 and I 4,[5],12:i:- pattern JPXX01.0026 were not observed in our sample of food animal *Salmonella,* they have repeatedly been observed in *Salmonella* recovered from chicken (PulseNet data; searches of the PulseNet national *Salmonella* database were conducted on 2012-06-11 and included data uploaded between 2008-06-12 and 2012-06-11). The absence of these patterns in *Salmonella* recovered from food animals in the northeast may simply reflect the limited amount of testing during the study period. Alternatively, the food responsible for illness may have come from outside the northeastern U.S., as some of the patterns have been observed in food animals from other areas in the U.S. (data not shown).

### Limitations

This study has several limitations. The first is the absence of data for testing food animal *Salmonella* isolates with a second-enzyme (BlnI). Testing human *Salmonella* isolates with BlnI has helped to discriminate within most PFGE patterns produced with XbaI. Beginning in 2011, VetNet specimens have been tested with BlnI, so this information will be available for future studies. A second limitation is the reduced number of samples collected by FSIS for *Salmonella* testing at federally inspected slaughter and processing plants in the northeast in recent years. A third limitation is the fact that only 11% of the human *Salmonella* isolates with the 16 common shared PFGE patterns were tested for susceptibility to antimicrobial agents. Finally, we recognize that not all food slaughtered and processed in a particular geographic region is consumed in the same region and that different regions of the U.S. vary in their poultry production concentrations.

## Conclusions

This work shows that 16 of the 20 most common PFGE patterns found in human *Salmonella* in Pennsylvania were also found in *Salmonella* recovered from food animals sampled at federally inspected slaughter and processing facilities in the northeastern U.S. Multidrug resistance was correlated with five PFGE patterns shared by food animal and human *Salmonella*: Berta pattern JAXX01.0001 (JAXX01.0003 ARS), Heidelberg pattern JF6X01.0022 (JF6X01.0015 ARS), Typhimurium patterns JPXX01.0003 (JPXX01.0003 ARS) and JPXX01.0018 (JPXX01.0002 ARS), and I 4,[5],12:i:- pattern JPXX01.0621 (TERX01.0001 ARS). The most common human pattern, *S*. Enteritidis patterns JEGX01.0004 (JEGX01.0003 ARS), accounting for 16% of all human *Salmonella*, was also common in *Salmonella* recovered from food animals, especially chicken. Our findings suggest an association between the most prevalent forms of human salmonellosis and contaminated meat and poultry. They also show a persistence of Enteritidis pattern JEGX01.0004 (JEGX01.0003 ARS), the association of differences in invasiveness with different PFGE patterns, and the tendency for some but not all PFGE subtypes within a serotype to acquire multidrug resistance. Comparing the PFGE fingerprint patterns and antimicrobial susceptibility profiles of *Salmonella* from humans with those from food animals inspected in slaughter and processing facilities located in a specific geographic region provides information that may assist in source attribution and outbreak investigations.

## References

[1] Scallan E, Hoekstra RM, Angulo FJ, Tauxe RV, Widdowson MA, et al (2011) Foodborne illness acquired in the United States–major pathogens. Emerg Infect Dis 17: 7–15. 21192848 10.3201/eid1701.P11101 PMC3375761

[2] Hoffmann M, Luo Y, Lafon PC, Timme R, Allard MW, et al.. (2013) Genome sequences of Salmonella enterica serovar Heidelberg isolates isolated in the United States from a multistate outbreak of human Salmonella infections. Genome announcements 1. 10.1128/genomeA.00004-12 PMC3569330 23405335

[3] Scharff RL (2012) Economic Burden from Health Losses Due to Foodborne Illness in the United States. Journal of Food Protection 75: 123–131. 22221364 10.4315/0362-028X.JFP-11-058

[4] Swaminathan B, Barrett TJ, Hunter SB, Tauxe RV (2001) PulseNet: the molecular subtyping network for foodborne bacterial disease surveillance, United States. Emerg Infect Dis 7: 382–389. 11384513 10.3201/eid0703.010303 PMC2631779

[5] Gerner-Smidt P, Hise K, Kincaid J, Hunter S, Rolando S, et al (2006) PulseNet USA: a five-year update. Foodborne Pathog Dis 3: 9–19. 16602975 10.1089/fpd.2006.3.9

[6] National Antimicrobial Resistance Monitoring System-FDA http://www.fda.gov/AnimalVeterinary/SafetyHealth/AntimicrobialResistance/NationalAntimicrobialResistanceMonitoringSystem/default.htm.Food and Drug Administration. Accessed 2013 September 19.

[7] Jackson CR, Fedorka-Cray PJ, Wineland N, Tankson JD, Barrett JB, et al (2007) Introduction to United States Department of Agriculture VetNet: status of Salmonella and Campylobacter databases from 2004 through 2005. Foodborne Pathog Dis 4: 241–248. 17600492 10.1089/fpd.2006.0067

[8] National Antimicrobial Resistance Monitoring System-USDA http://www.ars.usda.gov/Main/docs.htm?docid=6750.United States Department of Agriculture. Accessed 2013 September 19.

[9] Ribot EM, Fair MA, Gautom R, Cameron DN, Hunter SB, et al (2006) Standardization of pulsed-field gel electrophoresis protocols for the subtyping of Escherichia coli O157:H7, Salmonella, and Shigella for PulseNet. Foodborne Pathog Dis 3: 59–67. 16602980 10.1089/fpd.2006.3.59

[10] Brenner F, McWhorter-Murlin A (1998) Identification and serotyping of Salmonella and an update of the Kauffman-White scheme. Atlanta: Centers for Disease Control and Prevention.

[11] Farmer III J (1995) Enterobacteriaceae: Introduction and Identification In: Manual of Clinical Microbiology 6 th ed./editor in chief, P. Murray editors: Baron E, Pfaller M, Tenover F, Yolken R American Society for Microbiology Washington DC.

[12] USDA. National Antimicrobial Resistance Monitoring System – Enteric Bacteria AAN (2012) 2010 NARMS Animal Arm Annual Report. Athens, GA: U.S. Department of Agriculture, Agricultural Research Service.

[13] Clinical Laboratory Standards Institute W, Pennsylvania, (2010) Performance Standards for Antimicrobial Susceptibility Testing; Twentieth Informational Supplement. CLSI-document M100-S20. Clinical Laboratory Standards Institute.

[14] Centers for Disease Control and Prevention (2012) National Antimicrobial Resistance Monitoring System for Enteric Bacteria (NARMS): Human Isolates Final Report, 2010. Atlanta: Centers for Disease Control.

[15] Vugia DJ, Samuel M, Farley MM, Marcus R, Shiferaw B, et al (2004) Invasive Salmonella infections in the United States, FoodNet, 1996–1999: incidence, serotype distribution, and outcome. Clin Infect Dis 38 Suppl 3S149–156. 15095184 10.1086/381581

[16] Dean AG, Sullivan KM, Soe MM (2013) OpenEpi: Open Source Epidemiologic Statistics for Public Health, Version 3.01. http://www.openepi.com/v37/Menu/OE_Menu.htm. updated 2013/04/06, accessed 2013/08/22. Accessed 2013 September 19.

[17] Adaska JM, Silva AJ, Berge ACB, Sischo WM (2006) Genetic and Phenotypic Variability among Salmonella enterica Serovar Typhimurium Isolates from California Dairy Cattle and Humans. Applied and Environmental Microbiology 72: 6632–6637. 17021214 10.1128/AEM.01038-06 PMC1610315

[18] Woo YK, Lee SH (2006) Genetic diversity of multi-resistant Salmonella enterica serotype Typhimurium isolates from animals and humans. J Microbiol 44: 106–112. 16554725

[19] Soyer Y, Alcaine SD, Schoonmaker-Bopp DJ, Root TP, Warnick LD, et al (2010) Pulsed-field gel electrophoresis diversity of human and bovine clinical Salmonella isolates. Foodborne Pathog Dis 7: 707–717. 20180633 10.1089/fpd.2009.0424 PMC3132108

[20] Zou M, Keelara S, Thakur S (2012) Molecular characterization of Salmonella enterica serotype Enteritidis isolates from humans by antimicrobial resistance, virulence genes, and pulsed-field gel electrophoresis. Foodborne Pathog Dis 9: 232–238. 22283616 10.1089/fpd.2011.1012

[21] Centers for Disease Control and Prevention (2013) Surveillance for Foodborne Disease Outbreaks – United States, 2009–2010. Morbidity and Mortality Weekly Report 63: 41–56. PMC4604871 23344696

[22] Painter JA HR, Ayers T, Tauxe RV, Braden CR, Angulo FJ, et al. (2013) Attribution of foodborne illnesses, hospitalizations, and deaths to food commodities by using outbreak data, United States, 1998–2008. Emerg Infect Dis 19. 10.3201/eid1903.111866 PMC3647642 23622497

[23] Altekruse SF, Bauer N, Chanlongbutra A, DeSagun R, Naugle A, et al (2006) Salmonella enteritidis in broiler chickens, United States, 2000–2005. Emerg Infect Dis 12: 1848–1852. 17326935 10.3201/eid1212.060653 PMC3291361

[24] Kimura AC, Reddy V, Marcus R, Cieslak PR, Mohle-Boetani JC, et al (2004) Chicken consumption is a newly identified risk factor for sporadic Salmonella enterica serotype Enteritidis infections in the United States: a case-control study in FoodNet sites. Clin Infect Dis 38 Suppl 3S244–252. 15095196 10.1086/381576

[25] Marcus R, Varma JK, Medus C, Boothe EJ, Anderson BJ, et al (2007) Re-assessment of risk factors for sporadic Salmonella serotype Enteritidis infections: a case-control study in five FoodNet Sites, 2002–2003. Epidemiol Infect 135: 84–92. 16756692 10.1017/S0950268806006558 PMC2870546

[26] Currie A, MacDougall L, Aramini J, Gaulin C, Ahmed R, et al (2005) Frozen chicken nuggets and strips and eggs are leading risk factors for Salmonella Heidelberg infections in Canada. Epidemiology and infection 133: 809–816. 16181499 10.1017/S0950268805004383 PMC2870310

[27] Centers for Disease Control and Prevention (2010) Investigation update: multistate outbreak of human Salmonella Enteritidis infections associated with shell eggs Atlanta, Georgia. US Department of Health and Human Services, CDC.

[28] Allard MW, Luo Y, Strain E, Pettengill J, Timme R, et al (2013) On the Evolutionary History, Population Genetics and Diversity among Isolates of Salmonella Enteritidis PFGE Pattern JEGX01. 0004. PloS one 8: e55254. 23383127 10.1371/journal.pone.0055254 PMC3559427

[29] Food and Drug Administration US (2009) Prevention of Salmonella Enteritidis in shell eggs during production, storage, and transportation; final rule. Fed Regist. 33030–33101. 19588581

[30] Anonymous (2012) Annual Report on Zoonoses in Denmark 2011. National Food Institute, Technical University of Denmark.

[31] Wegener HC (2010) Danish initiatives to improve the safety of meat products. Meat Science 84: 276–283. 20374786 10.1016/j.meatsci.2009.06.025

[32] O’Brien SJ (2012) The “Decline and Fall” of Nontyphoidal Salmonella in the United Kingdom. Clin Infect Dis cis967 first published online November 19, 2012 doi:101093/cid/cis967. 10.1093/cid/cis967 PMC3563394 23166188

[33] Dechet AM, Scallan E, Gensheimer K, Hoekstra R, Gunderman-King J, et al (2006) Outbreak of multidrug-resistant Salmonella enterica serotype Typhimurium Definitive Type 104 infection linked to commercial ground beef, northeastern United States, 2003–2004. Clin Infect Dis 42: 747–752. 16477547 10.1086/500320

